# Glomerular lipidosis as a feature of renal-limited macrophage activation syndrome in a transplanted kidney: a case report

**DOI:** 10.1186/s12882-023-03380-2

**Published:** 2023-11-07

**Authors:** Kentaro Sugisaki, Takahiro Uchida, Sachiko Iwama, Masaaki Okihara, Isao Akashi, Yu Kihara, Osamu Konno, Masayuki Kuroda, Junki Koike, Hitoshi Iwamoto, Takashi Oda

**Affiliations:** 1 Department of Nephrology and Blood Purification, Kidney Disease Center, Tokyo Medical University Hachioji Medical Center, 1163 Tatemachi, Hachioji, Tokyo 193-0998 Japan; 2 Department of Kidney Transplantation Surgery, Kidney Disease Center, Tokyo Medical University Hachioji Medical Center, Hachioji, Tokyo Japan; 3 Center for Advanced Medicine, Chiba University, Chiba City, Chiba Japan; 4 Department of Pathology, St. Marianna University School of Medicine, Kawasaki, Kanagawa Japan

**Keywords:** CD68, CD8, Glomerular lipidosis, Macrophage activation syndrome (MAS), Histiocyte, Kidney transplant, Type V hyperlipidemia

## Abstract

**Background:**

Glomerular lipidosis is a rare histological feature presenting the extensive glomerular accumulation of lipids with or without histiocytic infiltration, which develops under various conditions. Among its various etiologies, macrophage activation syndrome (MAS) is a condition reported to be associated with histiocytic glomerular lipidosis. Here we describe the first case of glomerular lipidosis observed in a renal allograft that histologically mimicked histiocytic glomerulopathy owing to MAS.

**Case presentation:**

A 42-year-old man underwent successful living-donor kidney transplantation. However, middle-grade proteinuria and increased serum triglyceride levels indicative of type V hyperlipidemia developed rapidly thereafter. An allograft biopsy performed 6 months after the transplantation showed extensive glomerular infiltration of CD68^+^ foam cells (histiocytes) intermingled with many CD3^+^ T-cells (predominantly CD8^+^ cells). Furthermore, frequent contact between glomerular T-cells and histiocytes, and the existence of activated CD8^+^ cells (CD8^+^, HLA-DR^+^ cells) were observed by double immunostaining. There was no clinicopathological data suggesting lipoprotein glomerulopathy or lecithin cholesterol acyltransferase deficiency, both of which are well-known causes of glomerular lipidosis. The histological findings were relatively similar to those of histiocytic glomerulopathy caused by MAS. As systemic manifestations of MAS, such as fever, pancytopenia, coagulation abnormalities, hyperferritinemia, increased liver enzyme levels, hepatosplenomegaly, and lymphadenopathy were minimal, this patient was clinicopathologically diagnosed as having renal-limited MAS. Although optimal treatment strategies for MAS in kidney transplant patients remains unclear, we strengthened lipid-lowering therapy using pemafibrate, without modifying the amount of immunosuppressants. Serum triglyceride levels were normalized with this treatment; however, the patient’s extensive proteinuria and renal dysfunction did not improve. Biopsy analysis at 1 year after the transplantation demonstrated the disappearance of glomerular foamy changes, but the number of glomerular infiltrating cells remained similar.

**Conclusion:**

To our knowledge, this is the first reported case of glomerular lipidosis in a transplanted kidney. Increased interaction-activation of histiocytes (macrophages) and CD8^+^ T-cells, the key pathogenic feature of MAS, was observed in the glomeruli of this patient, who did not demonstrate overt systemic manifestations, suggesting a pathological condition of renal-limited MAS. The clinical effects of triglyceride-lowering therapy were limited, suggesting that hypertriglyceridemia was not the cause of but rather may be a consequence of renal-limited MAS.

**Supplementary Information:**

The online version contains supplementary material available at 10.1186/s12882-023-03380-2.

## Background

The diffuse and global foamy appearance of glomeruli, also called glomerular lipidosis, occurs as 2 different general lesions, namely, histiocytic and nonhistiocytic glomerular lesions. Histiocytic lesions are characterized by dilated glomerular capillary loops that contain lipid-laden histiocytes (foam cells). In contrast, a similar appearance of diffuse and global glomerular foamy changes without histiocytic infiltration is observed in several genetic and/or acquired enzymatic disorders, such as Fabry’s disease, lecithin-cholesterol acyltransferase (LCAT) deficiency, and lipoprotein glomerulopathy (LPG) [1–4]. Immunostaining for CD68 differentiates the diseases into 2 general categories; i.e., histiocytic and nonhistiocytic glomerular lipidosis.

A recent study reported typical cases of patients with vacuolated glomerular capillary loops caused by 5 different etiologies [5]. Herein, we report a case of glomerular lipidosis in a transplanted kidney. Our case histologically mimicked histiocytic glomerulopathy secondary to macrophage activation syndrome (MAS) among the 5 reported types [5].

The term MAS originally referred to a hemophagocytic syndrome (HPS) variant that occurs in patients with autoinflammatory and autoimmune diseases, particularly systemic onset juvenile rheumatoid arthritis and adult-onset Still’s disease. However, in recent reports on HPS in adults, the term MAS is often used almost interchangeably with acquired HPS. Moreover, there is no consensus at present regarding the nomenclature for HPS- and MAS-associated diseases [6, 7], and the histological presence of hemophagocytosis is sometimes difficult to detect, particularly in the initial stages, so some classification criteria of MAS do not include the presence of hemophagocytosis as a diagnostic item [8]. Therefore, we use the term MAS or HPS/MAS throughout this report to describe the general condition of HPS/MAS. Although the precise pathogenesis is unknown, the uncontrolled activation/proliferation of nonmalignant macrophages, leading to aberrant antigen presentation to CD8^+^ T-cells is suspected to be an essential factor. This causes aberrant cytokine release from these cells, leading to tissue damage [9, 10].

Recently, a report on 4 cases of patients with renal-limited HPS/MAS was published. All patients presented with histiocytic glomerulopathy, but did not fulfill the current classification criteria for systemic HPS/MAS [11, 12], suggesting the existence of clinical conditions associated with HPS/MAS that can only be diagnosed by renal biopsy. Santoriello et al. reported a similar case of a patient with glomerulopathy and intraglomerular hemophagocytosis [13].

Although renal transplantation is common for patients with systemic HPS/MAS [14, 15], there are no reports to date on the histological features of the renal allografts of patients with HPS/MAS. Here, we describe a patient who developed severe hypertriglyceridemia and proteinuria soon after kidney transplantation, and this is the first case to our knowledge of glomerular lipidosis in a renal allograft. The histology of the kidney mimicked the reported features of histiocytic glomerulopathy secondary to MAS [5, 13] but with minor systemic clinical manifestations, and the patient was ultimately diagnosed as having renal-limited MAS.

## Case presentation

A 42-year-old Japanese man was admitted to our hospital for a kidney transplant. According to the referral form, he had no medical history except for hypertension that had been left untreated for a decade. He had bilateral pretibial edema and exacerbating respiratory distress for one month. When he visited a nearby hospital, hemodialysis initiation was necessary as his kidney function was terminal (serum creatinine: 14.8 mg/dL) and he had severe dyspnea. The patient had 3 + proteinuria (8.36 g/gCre) and 1 + urinary occult blood. Serum laboratory data showed that his albumin level was 2.9 g/dL, and he was negative for the anti-neutrophil cytoplasmic antibody, anti-glomerular basement membrane antibody, and anti-nuclear antibody. Abnormalities in complement and immunoglobulin levels were not observed. Renal biopsy showed widespread collapsed glomeruli (8/8), and immunofluorescence microscopy (IF) revealed negative results, and electron microscopy (EM) demonstrated minor changes. The final diagnosis was acute kidney injury associated with minor glomerular abnormalities (MGA) accompanied with chronic histological changes caused by untreated hypertension. As the patient did not recover from dialysis-dependent renal failure, he was referred for kidney transplantation.

**Fig. 1:**
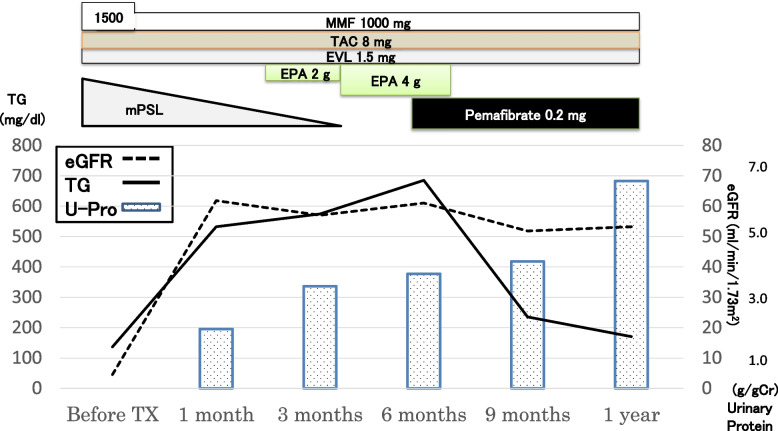
Clinical course of the patient. Induction therapy for kidney transplantation was performed using methylprednisolone (mPSL), tacrolimus (TAC), mycophenolate mofetil (MMF), and everolimus (EVL). Soon after kidney transplantation, triglyceride levels increased together with an increase in proteinuria. At 6 months after the transplantation, intensive lipid-lowering therapy using pemafibrate was started. One year after the transplantation, the patient’s renal function and proteinuria had not improved

At initial admission, his body weight was 80.0 kg (BMI 27), and blood pressure was 127/88 mmHg. Dyslipidemia and diabetes mellitus were not observed (triglyceride level: 136 mg/dL, and HbA1c 4.7%). Five months later, a living-donor kidney transplant was performed with his mother as the donor. His clinical course is shown in Fig. 1. Induction therapies for transplantation included methylprednisolone, tacrolimus, mycophenolate mofetil, and everolimus. A kidney biopsy performed 0 h after transplantation showed MGA (data not shown). The transplantation was successful, and laboratory data at the time of discharge were urinary protein 1 + (1.02 g/gCre), urinary occult blood 1 + , and serum creatinine 1.07 mg/dL.

In the first month after transplantation, his urinary protein level increased to 1.95 g/gCre, with an increase in triglyceride level to 552 mg/dL. Three months after transplantation, a protocol biopsy was performed (Supplementary Figure S1). The formalin-fixed paraffin-embedded tissue sections contained 24 glomeruli, 3 of which showed global sclerosis but no crescents. IF findings were negative. The overall histological diagnosis was MGA; however, immunohistochemical analysis revealed mild to moderate glomerular infiltration of CD3^+^, CD8^+^, and CD68^+^ cells. At this point, his urinary protein level further increased to 3.36 g/gCre, but there was no deterioration in renal function (Fig. 1).

**Fig. 2:**
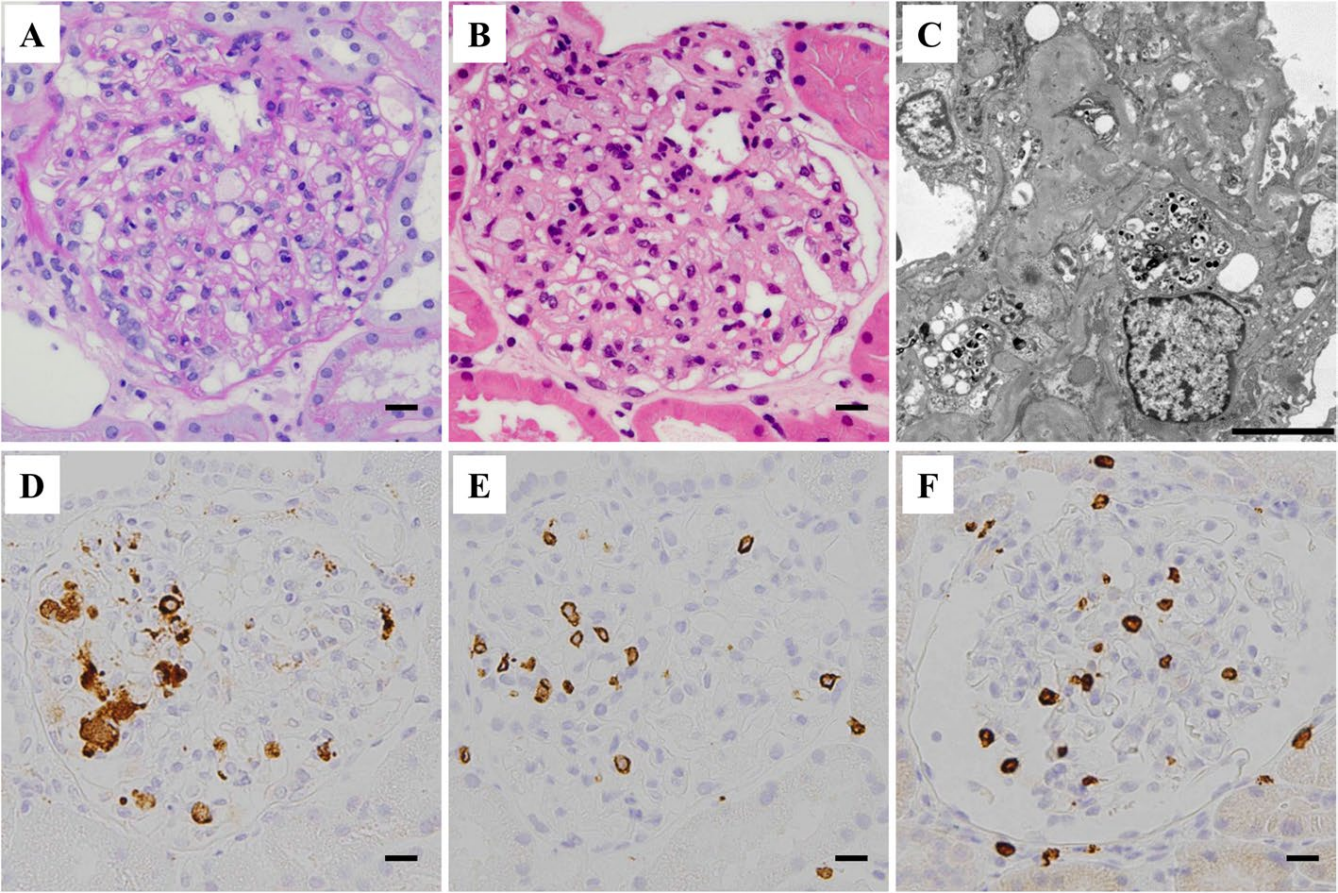
Histological features of the renal biopsy performed 6 months after the transplantation. **A**, **B** Light microscopy image showing numerous foam cells within the glomerular capillaries (**A**, PAS; **B**, HE; scale bars = 10.0 μm). **C** Electron microscopy image showing foam cells in a glomerulus. Scale bar = 5.0 μm. **D**, **E**, **F** Immunoperoxidase staining images showing extensive glomerular accumulation of vacuolated CD68 (clone: KP1)^+^ cells (**D**), intermingled with CD3^+^ cells (**E**), most of which are positive for CD8 (**F**). Scale bars = 10.0 μm

**Fig. 3:**
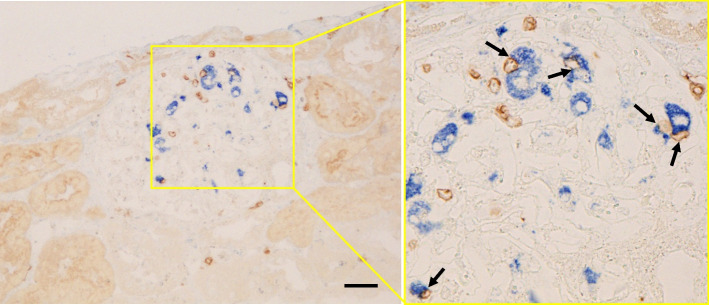
Double immunostaining for CD68 (clone: PG-M1; alkaline phosphatase, blue) and CD3 (peroxidase, brown) in the renal biopsy tissue performed 6 months after the transplantation. Extensive glomerular accumulation of CD68^+^/CD3^+^ cells is shown (scale bar = 20.0 μm), and on higher magnification, close contact between these cells is demonstrated (arrows)

**Fig. 4:**
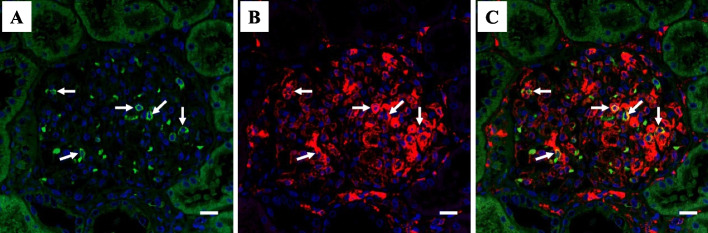
Double immunofluorescence staining for CD8 (**A**; Alexa Fluor 488, green) and HLA-DR (**B**; Alexa Fluor 594, red), together with DAPI (blue) nuclear staining in the renal biopsy tissue performed 6 months after the transplantation. The merged image demonstrated the existence of some double-positive cells (**C**; yellow cells, indicated by arrows) within glomeruli. Scale bar = 10.0 μm

Six months after the transplantation, his kidney function was still stable; however, his triglyceride level increased further to 685 mg/dL, along with a decrease in high-density lipoprotein-cholesterol levels. The patient’s serum lipid profile is summarized in Table 1. LCAT activity was normal. A substantial increase in triglycerides together with an increase in the lipoprotein fraction of pre-β (very-low-density lipoprotein [VLDL]-cholesterol) and a decrease in lipoprotein lipase (LPL) activity were observed. These findings suggested that the patient has asymptomatic type V hyperlipidemia. The HbA1c was elevated to 7.5% at this time but improved under 7.0% immediately after induction of dipeptidyl peptidase-4 inhibitor. Soluble interleukin-2 receptor (sIL-2R) and CRP levels were relatively high at 500 U/mL and 1.13 mg/dL, respectively, suggesting some underlying inflammatory status. However, there were no serologic findings suggestive of specific viral infection (Table 1). A renal biopsy was performed again, which contained 21 glomeruli of which 5 showed global sclerosis and no crescents. The remaining glomeruli showed the characteristic diffuse feature of extensive accumulation of foam cells (Fig. 2). IF findings were all negative (data not shown). Under EM, many glomerular foam cells were observed, but there were no electron-dense deposits. Immunoperoxidase staining revealed that cells with a foamy appearance in the capillary loops were positive for CD68, and there was extensive infiltration of CD3^+^ T-cells (predominantly CD8^+^ cells) (Fig. 2, Supplementary Figure S2). Further analysis using double immunostaining revealed frequent contact between these cells (Fig. 3), and the existence of activated (HLA-DR^+^) CD8^+^ T-cells (Fig. 4).

**Table 1:** Laboratory data and lipid profile at six months after transplantation

**Test items**		**Lipid profiles**			**reference range**
**Hematology**		Triglyceride (mg/dL)		685	40–234
**WBC (/μL)**	6390	Total cholesterol (mg/dL)		222	142–248
**Hb (g/dL)**	11.0	LDL cholesterol (mg/dL)		90.6	65–163
**Platelets (/μL)**	25.3	HDL cholesterol (mg/dL)		33.0	38–90
**Serum chemistry**		Apo A-I (mg/dl)		106	119–155
**AST (IU/L)**	21	Apo A-II (mg/dl)		21.3	25.9–35.7
**ALT (IU/L)**	14	Apo B (mg/dl)		110	73–109
**Bilirubin (IU/L)**	0.4	Apo C-II (mg/dl)		12.9	1.8–4.6
**LDH (IU/L)**	174	Apo C-III (mg/dl)		24.2	5.8–10.0
**Ferritin (ng/ml)**	376.8	Apo E (mg/dl)		9.9	2.7–4.3
**HbA1c (%)**	7.5	Lipoprotein fraction (%)			
**TP (g/dL)**	7.8	α		15.8	26.9–50.5
**Alb (g/dL)**	3.9	Pre-β		43.2	7.9–23.8
**Creatinine (mg/dL)**	1.12	Β		30.1	35.3–55.5
**CRP (mg/dL)**	1.13	CETP (mg/dL)		162	90–200
**Proteinuria (g/gCre)**	3.77	E/T ratio (%)		68	73–77
**sIL-2R (U/ml)** **(reference range 184–486)**	500	Sd LDL (mg/dL)		47.8	9.5–42.5
		LPL activity		25	164–284
**Viral tests**					
**EBV VCA IgM (EIA) (reference range < 0.5)**			0.5 ( ±)		
**EBV VCA IgG (EIA) (reference range < 0.5)**			0.5 ( +)		
**EBV EBNA IgG (EIA) (reference range < 0.5)**			0.5 ( +)		
**CMV IgM (EIA) (reference range < 0.85)**			0.73 (˗)		
**CMV IgG (EIA) (reference range < 6.0)**			8.6 ( +)		
**C7-HRP (reference range: negative)**			Negative		
**Parvovirus B19 IgM (EIA) (reference range < 0.8)**			0.31 (˗)		

*sIL-2R* Soluble interleukin-2 receptor, *CETP* Cholesteryl ester transfer protein, *E/T* Ester/Total cholesterol, *Sd* Small dense, *LPL* Lipoprotein lipase, *LDL* Low density lipoprotein, *HDL* High density lipoprotein, *EBV* Epstein-Barr virus, *CMV* Cytomegalovirus

**Fig. 5:**
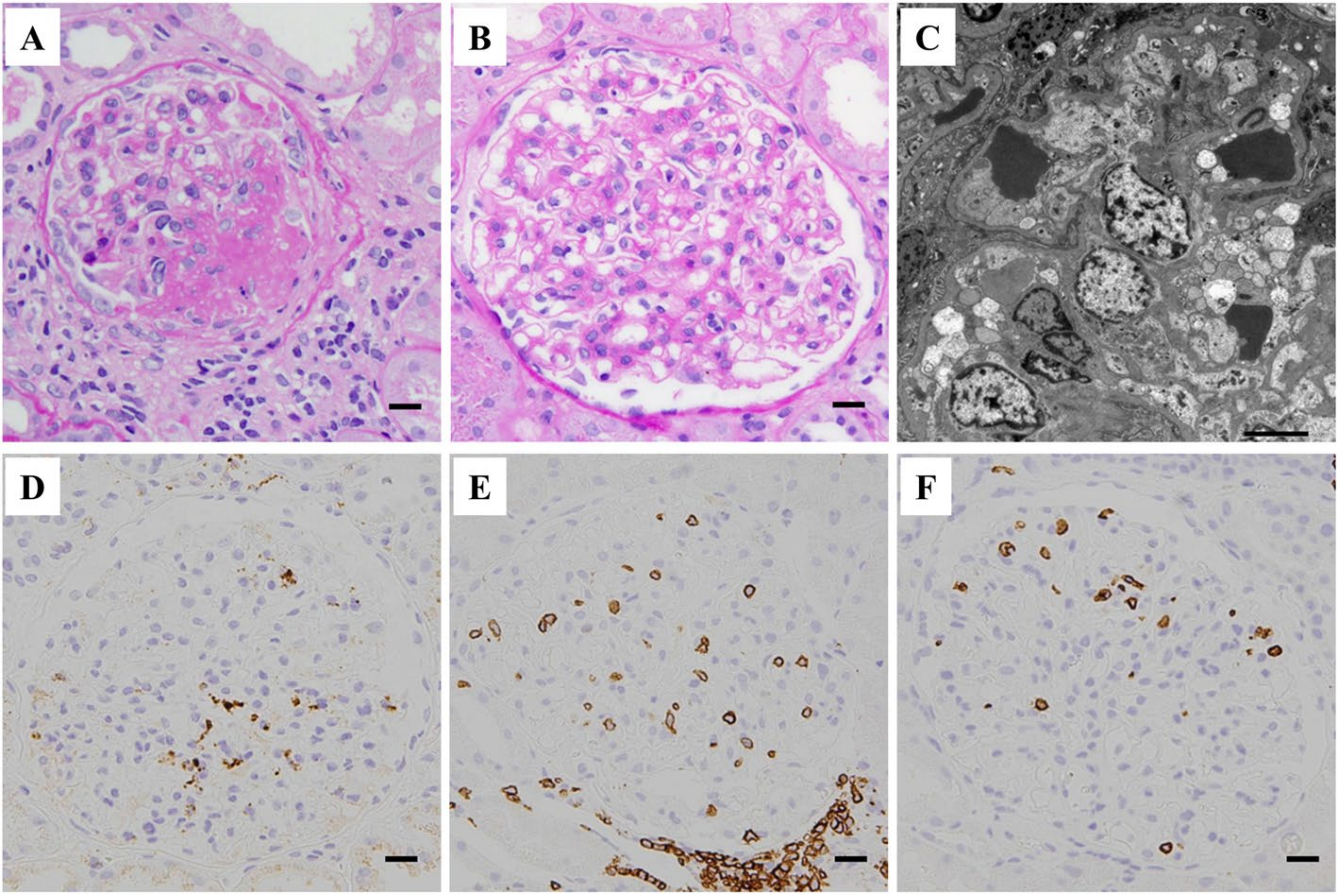
Histological features of the renal biopsy 1 year after the transplantation. Light microscopy image showing a glomerulus with typical segmental sclerosis (**A**, PAS staining; scale bar = 10.0 μm). In another glomerulus with minor changes, the disappearance of foam cells within the glomerular capillaries is shown (**B**, PAS staining; scale bar = 10.0 μm). **C** Electron microscopy image demonstrating minor glomerular abnormalities (scale bar = 5.0 μm). **D**, **E**, **F** Immunohistochemistry showing an apparent decrease in glomerular lipidosis, whereas the accumulation of cells positive for CD68 (clone: KP1; **D**), for CD3 (**E**), and for CD8 (**F**) were essentially unchanged (scale bars = 10.0 μm)

After the histological characteristics were clarified, intensive lipid-lowering therapy with pemafibrate was performed aiming at reducing the glomerular lipid deposition. Results of the protocol biopsy performed 1 year after transplantation are shown in Fig. 5. The 21 glomeruli showed no crescents, but 4 showed global sclerosis and 3 showed segmental sclerosis. Foamy changes were no longer observed in the glomerular capillary lumen. Mild interstitial fibrosis and tubular atrophy were observed. The overall clinicopathological diagnosis was secondary focal segmental glomerulosclerosis. Immunoperoxidase staining revealed an apparent decrease in lipid accumulation in CD68^+^ histiocytes; however, the number of CD8^+^ cells and CD68^+^ histiocytes remained at almost a similar level (Fig. 5, Supplementary Figure S3). Despite the significant decrease in triglyceride levels, proteinuria did not improve but was in a worsening trend between 9 months and 1 year after the transplantation with pemafibrate treatment (Fig. 1). Clinically, however, there were no changes in body weight or blood pressure during this period.

## Discussion and conclusions

Here, we presented the first case to our knowledge of glomerular lipidosis in a transplanted kidney associated with the development of type V hyperlipidemia. Histological analysis revealed extensive glomerular accumulation of CD68^+^ histiocytes and CD8^+^ T-cells, and interaction of these cells with the presence of activated CD8^+^ T-cells locally in glomeruli, which is a key pathologic characteristic of MAS.

Cases of glomerular lipidosis are rare and difficult to diagnose; the importance of EM observation has been indicated [4]. A previous study reported renal biopsy findings of 5 typical cases of patients with glomerular lipidosis with vacuolated glomerular capillary loops of different etiologies [5]; i.e., 3 histiocytic lesions (crystal-storing histiocytosis, histiocytic glomerulopathy, and thrombotic microangiopathy [TMA]), and 2 nonhistiocytic lesions (LCAT-deficiency and LPG). Interestingly, mutations in Apo E can result in histiocytic (Apo E2 homozygote glomerulopathy) and nonhistiocytic glomerulopathy (LPG), depending on the mutation types [3]. Our patient showed extensive accumulation of foamy CD68^+^ histiocytes within the glomerular capillary loops without crystals and TMA. Thus, the histological features of our case matched the histiocytic glomerulopathy secondary to MAS reported in a previous study [5]. Another study reported similar glomerulopathy as well as intraglomerular hemophagocytosis in association with HPS/MAS [13]. The possibility of glomerular lipidosis in renal allograft as a recurrent nephropathy was unlikely because there were no histological findings of glomerular lipidosis in the biopsy tissue of native kidney. However, the histology of the native kidney is so advanced that we cannot rule out the possibility that it was the tissue after the characteristic findings of glomerular lipidosis had already disappeared.

Typical patients with MAS develop a variety of systemic manifestations, such as fever, pancytopenia, coagulation abnormalities, hyperferritinemia, hypertriglyceridemia, increase in liver enzyme levels, hepatosplenomegaly, and lymphadenopathy. Our patient had severe hypertriglyceridemia that was obviously abnormal, but other manifestations were mild or absent. Mild abnormalities in laboratory data are possibly associated with MAS, including high levels of ferritin, LDH, and sIL-2R, as they do not fulfill the general diagnostic criteria of HPS/MAS [11].

Although the precise mechanism of hypertriglyceridemia in MAS needs to be elucidated, it is generally considered to occur as a consequence of a decrease in the activity of lipid-degrading enzymes owing to the inhibitory effect of inflammatory cytokines produced under the condition of MAS. This concept is supported by a previous study in diabetic rats demonstrating that the administration of tumor necrosis factor, which is an inflammatory cytokine, increases serum triglyceride and VLDL-cholesterol levels through the suppression of LPL activity, leading to the development of type V hyperlipidemia [16].

Although the precise pathogenesis of MAS requires further elucidation, it can be outlined as follows: patients with MAS have certain antigenic triggers and dysfunctions in cytotoxic T-cells and natural killer cells, both of which are involved in killing antigen-presenting cells (APCs), resulting in the sustained proliferation of APCs with sustained antigen presentation and the activation of CD8^+^ T-cells. Activated CD8^+^ T-cells aberrantly produce cytokines, which induces the uncontrolled activation of histiocytes and tissue damage [17]. Analyses of perforin-deficient mice (HPS/MAS model) also confirmed the essential role of CD8^+^ T-cells secreting IFN-γ in the development of HPS/MAS [10]. In the present patient, the accumulation and the frequent contact between CD8^+^ T-cells and CD68^+^ histiocytes, and the existence of activated (HLA-DR^+^) CD8^+^ T-cells were observed in the glomeruli (Figs. 3, 4), suggesting the ongoing occurrence of antigen presentation and immunological activation by these cells, similar to MAS, locally in the glomeruli.

Acquired HPS/MAS develops in patients with autoimmune diseases or certain infections [17]. Renal transplantation is common in patients with HPS/MAS [14, 15], attributed to coexisting infections in an immunosuppressive state. However, in the present patient, serological data did not suggest the existence of an autoimmune disease or an overt infection. Therefore, we suspected the existence of an unknown, asymptomatic viral infection, although we could not find out any serological evidence (Table 1: serological data on Epstein-Barr virus, cytomegalovirus, and parvovirus) or histological evidence for viral infection such as viral-like particles on electron microscopy.

The optimal treatment strategy for HPS/MAS in transplant patients remains unclear. Generally, HPS/MAS is treated with immunosuppressants; however, in transplant patients, HPS/MAS usually develops in the background of an infection attributed to immunosuppressants administered for the transplantation. Hence, it is difficult to increase the immunosuppressant dose. Moreover, in the present patient, HPS/MAS showed only renal-limited manifestations. As a previous report demonstrated the glomerular accumulation of CD68^+^ macrophages in hypertriglyceridemia mice compared with normal mice, suggesting an association between serum triglyceride levels and glomerular CD68^+^ cells [18], we used pemafibrate, a novel selective peroxisome proliferator-activated receptor (PPAR)-α modulator, in expectation of improving lipid metabolism, and reducing histiocytic accumulation. PPAR-α ligands are known to induce apoptosis in macrophages by negatively interfering with the apoptosis signaling pathway [19]. After the administration of pemafibrate without increasing the dose of immunosuppressants, we observed improvement in hypertriglyceridemia and a decrease in glomerular lipid deposition. However, the number of CD8^+^ and CD68^+^ cells within glomeruli remained at an almost similar level (Supplementary Figure S3), and severe proteinuria did not improve after the treatment. Such responsiveness to therapy suggested that hypertriglyceridemia (type V hyperlipidemia) might not be the cause but may rather be the consequence of renal limited MAS in the present case. As for steroids, we decided not to use them because his clinical symptoms were mild with mild decrease in serum albumin, and there were also concerns about exacerbating both the viral infection as possible etiology and secondary diabetes mellitus. The possibility that tacrolimus overdose contributed to renal dysfunction and severe proteinuria was unlikely, because serum levels of tacrolimus were measured and controlled at every visit, and there were no histological findings to suggest tissue damage caused by calcineurin inhibitors.

To our knowledge, this is the first reported case of glomerular lipidosis in a transplanted kidney as histiocytic glomerulopathy without systemic manifestations of MAS, but with the local interaction of CD68^+^ histiocytes and CD8^+^ T-cells, suggesting renal-limited MAS. Although glomerular manifestations occurred with the development of type V hyperlipidemia, treatment by lipid-lowering therapy was unable to improve the clinical manifestations of extensive proteinuria and renal dysfunction. We therefore suspect that hypertriglyceridemia was not an essential pathogenic factor but might have been an aggravation factor in this patient. In addition, histological analysis of glomerular infiltrating cells using various cell markers is an important tool in understanding the pathogenesis of glomerular lipidosis, as in this case.

### Supplementary Information

Below is the link to the electronic supplementary material.

**Additional file 1: Supplementary Figure S1.** Histological features of the renal biopsy performed 3 months after the transplantation. (A, B) Light microscopy image showing minor glomerular abnormalities (A, PAS; B, HE; scale bars = 10.0 μm). (C) Electron microscopy image showing minor glomerular abnormalities (scale bar = 5.0 μm). (D, E, F). Immunoperoxidase staining image showing mild to moderate but substantial glomerular accumulation of cells positive for CD68 (clone: KP1; D), for CD3 (E), and for CD8 (F) (scale bars = 10.0 μm).

**Additional file 2: Supplementary Figure S2.** Double immunofluorescence staining for CD8 (A; Alexa Fluor 488, green) and CD3 (B; Alexa Fluor 594, red), together with DAPI (blue) nuclear staining in the renal biopsy tissue performed 6 months after the transplantation. The merged image (C) demonstrated that the population of CD8^-^ CD3^+^ cells were minor (red cells, indicated by arrows) and most cells (more than 80%) were double positive for CD8 and CD3 (yellow~green cells) within glomeruli. Scale bar = 10.0 μm.

**Additional file 3: Supplementary Figure S3.** Changes in the number of cells positive for CD68 (clone: KP1), and for CD8 in the glomeruli of renal biopsies performed at 3 months, 6 months, and 1 year after transplantation. Cell counts for CD68 and CD8 were still high in the renal biopsy tissue after lipid-lowering therapy (at 1 year after transplantation).

## Data Availability

Not applicable.

## Abbreviations

HPS: Hemophagocytic syndrome
LCAT: Lecithin-cholesterol acyltransferase
LPG: Lipoprotein glomerulopathy
MAS: Macrophage activation syndrome
IF: Immunofluorescence microscopy
EM: Electron microcopy
MGA: Minor glomerular abnormality
VLDL: Very-low-density lipoprotein
LPL: Lipoprotein lipase
sIL-2R: Soluble interleukin-2 receptor
TMA: Thrombotic microangiopathy
APCs: ANTIGEN-presenting cells
PPAR: Peroxisome proliferator-activated receptor
