# Supplementary material for: Glomerular lipidosis as a feature of renal-limited macrophage activation syndrome in a transplanted kidney: a case report
Source: BMC Nephrol. 2023 Nov 7;24:329. doi: 10.1186/s12882-023-03380-2 (PMC10631159; doi:10.1186/s12882-023-03380-2)
Supplement: Supplementary file 1 — Additional file 1: Supplementary Figure S1. Histological features of the renal biopsy performed 3 months after the transplantation. (A, B) Light microscopy image showing minor glomerular abnormalities (A, PAS; B, HE; scale bars = 10.0 μm). (C) Electron microscopy image showing minor glomerular abnormalities (scale bar = 5.0 μm). (D, E, F). Immunoperoxidase staining image showing mild to moderate but substantial glomerular accumulation of cells positive for CD68 (clone: KP1; D), for CD3 (E), and for CD8 (F) (scale bars = 10.0 μm). [file 12882_2023_3380_MOESM1_ESM.pptx]

## Slide 1
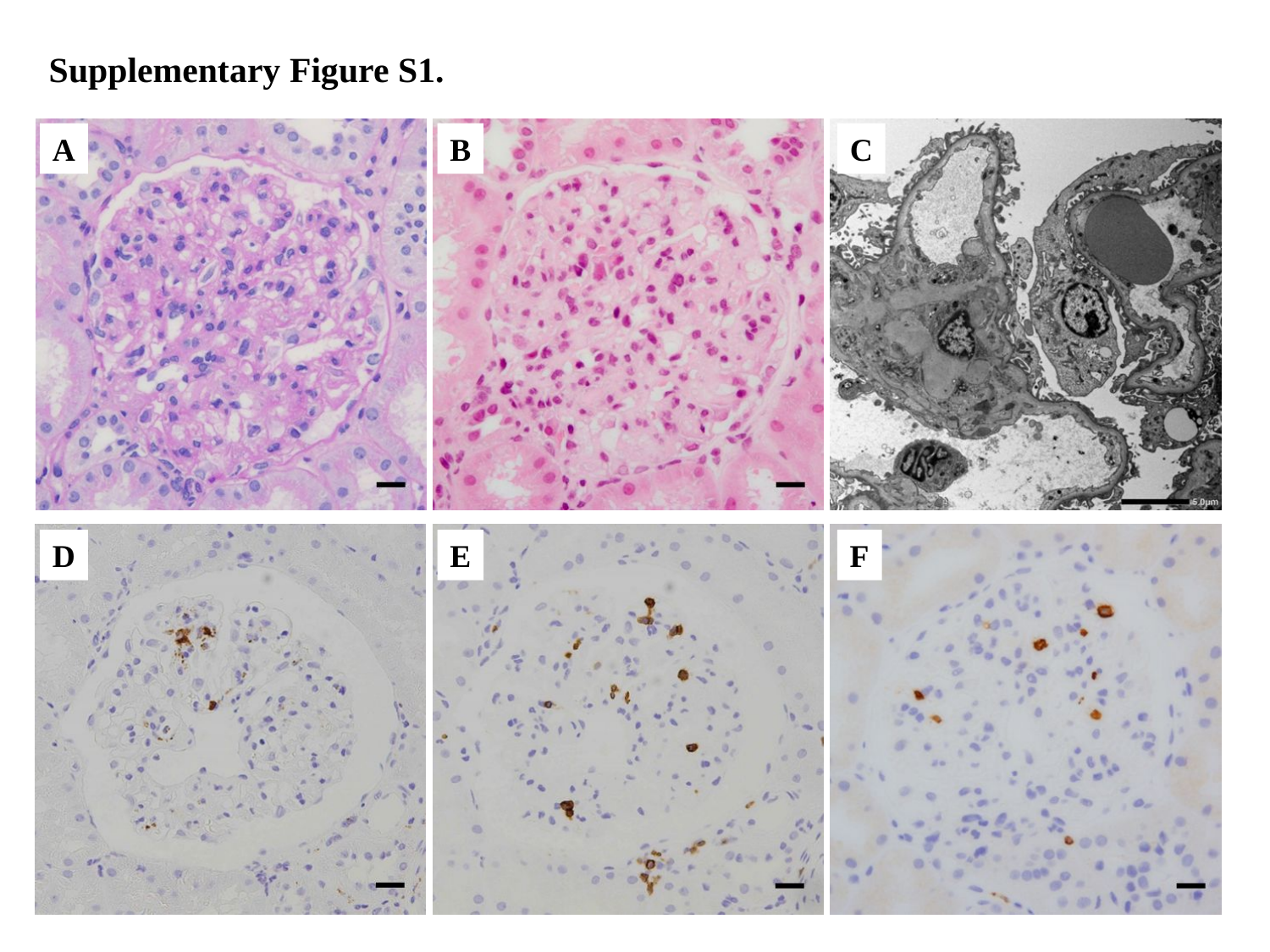

Supplementary Figure S1.
A
B
C
E
F
D

## Slide 2
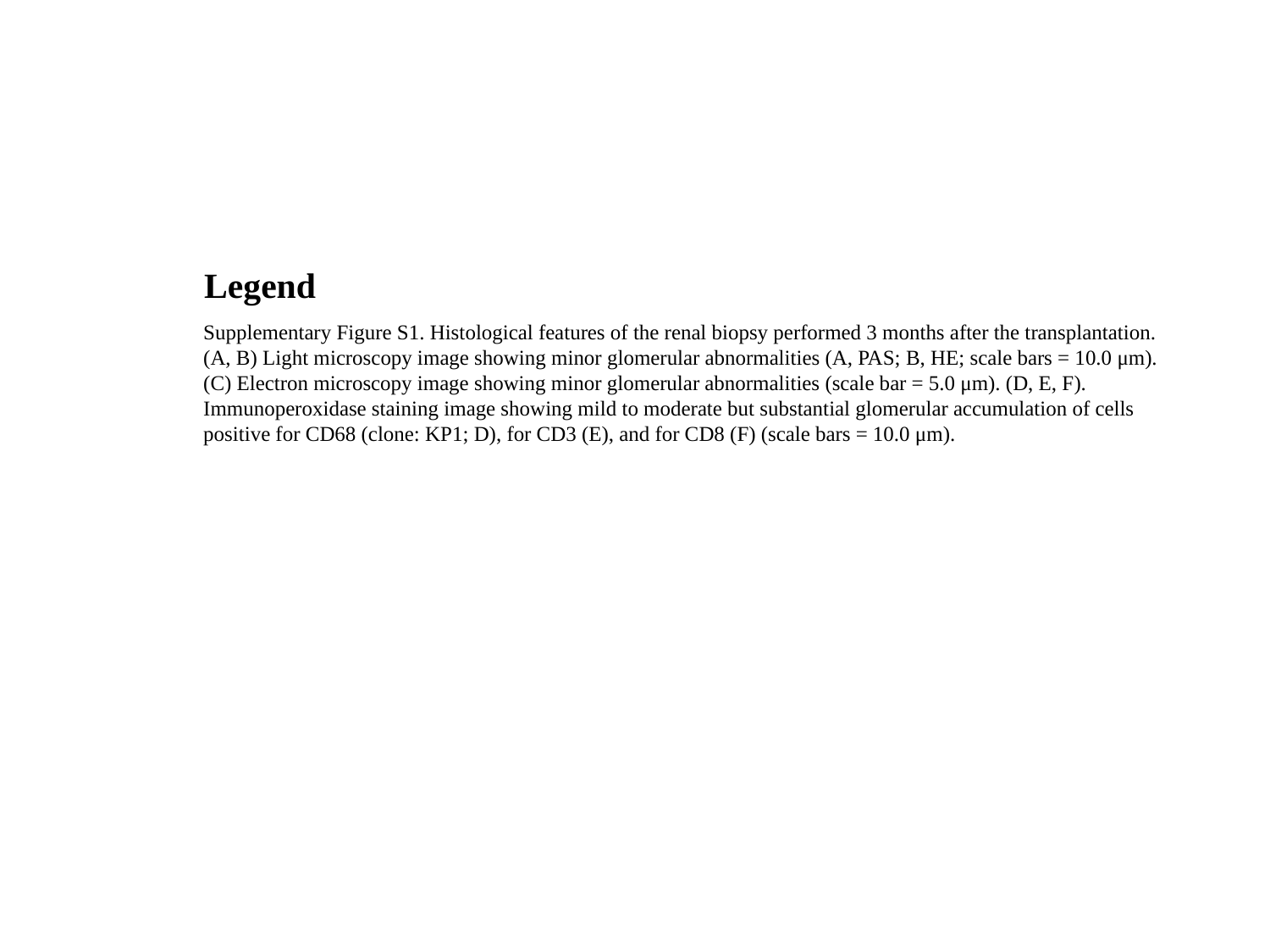

Legend
Supplementary Figure S1. Histological features of the renal biopsy performed 3 months after the transplantation. (A, B) Light microscopy image showing minor glomerular abnormalities (A, PAS; B, HE; scale bars = 10.0 μm). (C) Electron microscopy image showing minor glomerular abnormalities (scale bar = 5.0 μm). (D, E, F). Immunoperoxidase staining image showing mild to moderate but substantial glomerular accumulation of cells positive for CD68 (clone: KP1; D), for CD3 (E), and for CD8 (F) (scale bars = 10.0 μm).
