# Supplementary material for: Glomerular lipidosis as a feature of renal-limited macrophage activation syndrome in a transplanted kidney: a case report
Source: BMC Nephrol. 2023 Nov 7;24:329. doi: 10.1186/s12882-023-03380-2 (PMC10631159; doi:10.1186/s12882-023-03380-2)
Supplement: Supplementary file 2 — Additional file 2: Supplementary Figure S2. Double immunofluorescence staining for CD8 (A; Alexa Fluor 488, green) and CD3 (B; Alexa Fluor 594, red), together with DAPI (blue) nuclear staining in the renal biopsy tissue performed 6 months after the transplantation. The merged image (C) demonstrated that the population of CD8- CD3+ cells were minor (red cells, indicated by arrows) and most cells (more than 80%) were double positive for CD8 and CD3 (yellow~green cells) within glomeruli. Scale bar = 10.0 μm. [file 12882_2023_3380_MOESM2_ESM.pptx]

## Slide 1
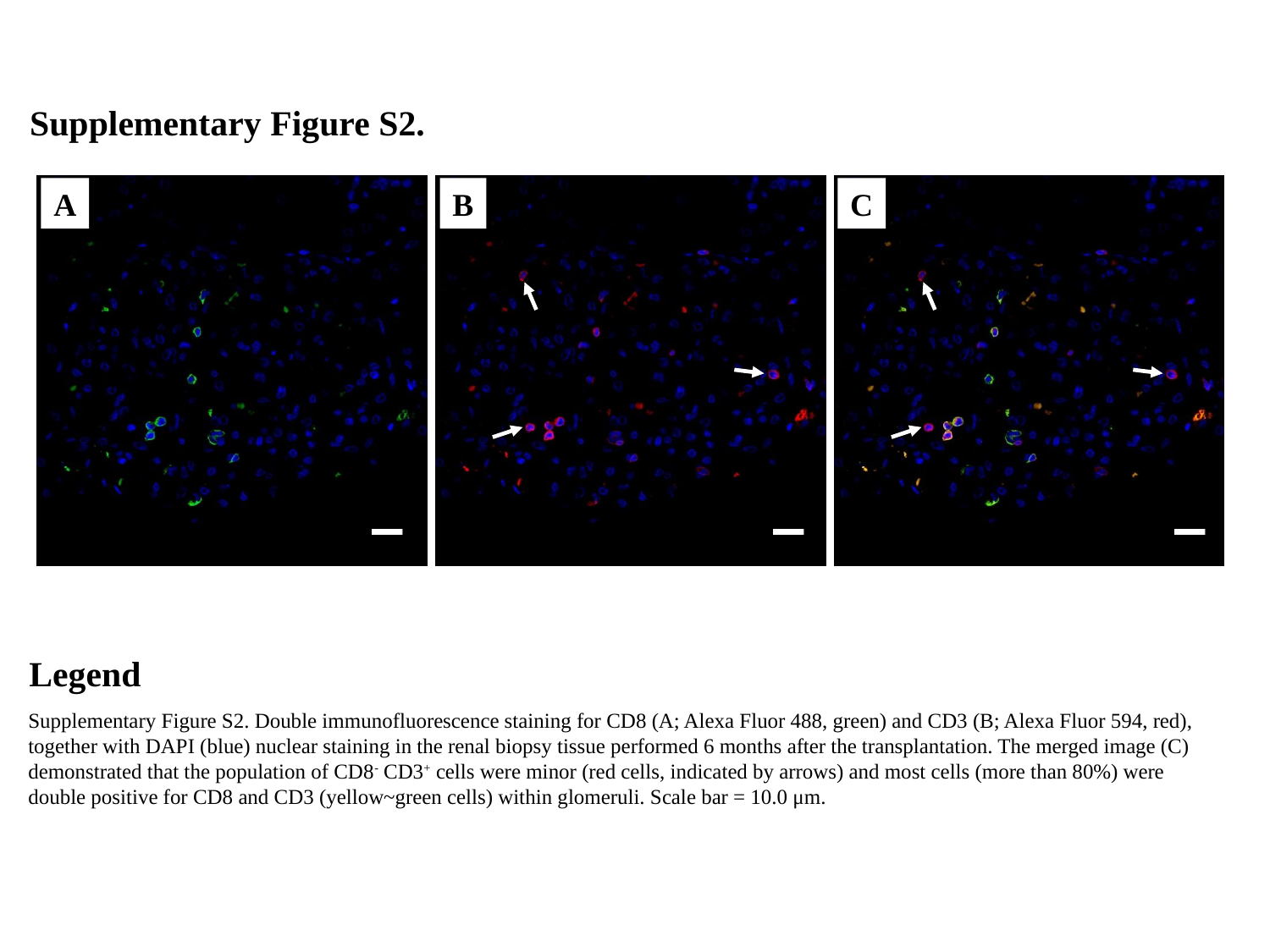

Supplementary Figure S2.
A
B
C
Legend
Supplementary Figure S2. Double immunofluorescence staining for CD8 (A; Alexa Fluor 488, green) and CD3 (B; Alexa Fluor 594, red), together with DAPI (blue) nuclear staining in the renal biopsy tissue performed 6 months after the transplantation. The merged image (C) demonstrated that the population of CD8- CD3+ cells were minor (red cells, indicated by arrows) and most cells (more than 80%) were double positive for CD8 and CD3 (yellow~green cells) within glomeruli. Scale bar = 10.0 μm.
