# Supplementary material for: Glomerular lipidosis as a feature of renal-limited macrophage activation syndrome in a transplanted kidney: a case report
Source: BMC Nephrol. 2023 Nov 7;24:329. doi: 10.1186/s12882-023-03380-2 (PMC10631159; doi:10.1186/s12882-023-03380-2)
Supplement: Supplementary file 3 — Additional file 3: Supplementary Figure S3. Changes in the number of cells positive for CD68 (clone: KP1), and for CD8 in the glomeruli of renal biopsies performed at 3 months, 6 months, and 1 year after transplantation. Cell counts for CD68 and CD8 were still high in the renal biopsy tissue after lipid-lowering therapy (at 1 year after transplantation). [file 12882_2023_3380_MOESM3_ESM.pptx]

## Slide 1
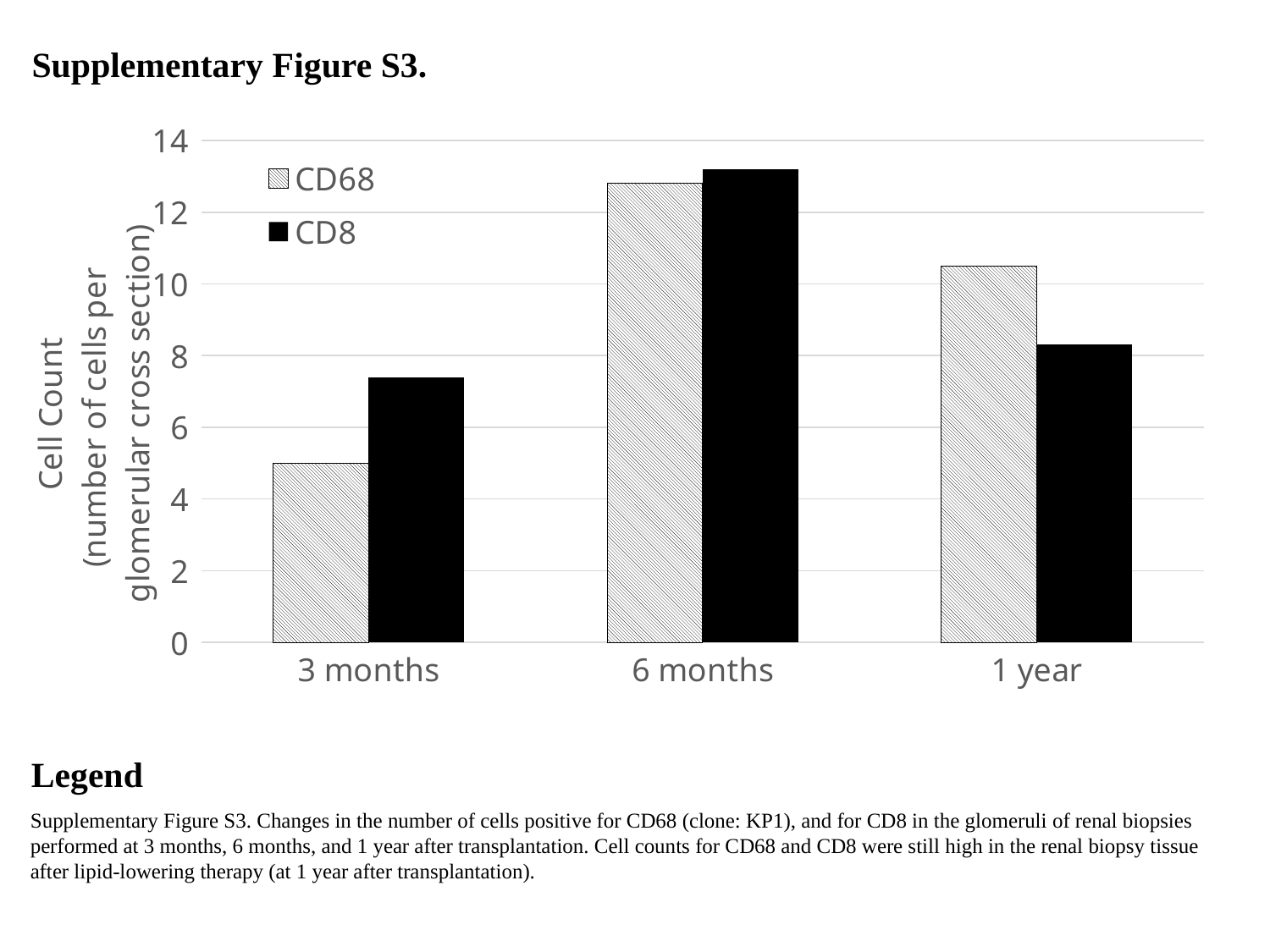

Supplementary Figure S3.
### Chart
| Category | CD68 | CD8 |
|---|---|---|
| 3 months | 5.0 | 7.4 |
| 6 months | 12.8 | 13.2 |
| 1 year | 10.5 | 8.3 |Legend
Supplementary Figure S3. Changes in the number of cells positive for CD68 (clone: KP1), and for CD8 in the glomeruli of renal biopsies performed at 3 months, 6 months, and 1 year after transplantation. Cell counts for CD68 and CD8 were still high in the renal biopsy tissue after lipid-lowering therapy (at 1 year after transplantation).
